# Meaning-in-Life Profiles among Chinese Late Adolescents: Associations with Readiness for Political Participation

**DOI:** 10.3390/ijerph18115765

**Published:** 2021-05-27

**Authors:** Li Lin, Daniel T. L. Shek

**Affiliations:** Department of Applied Social Sciences, The Hong Kong Polytechnic University, Hong Kong, China; daniel.shek@polyu.edu.hk

**Keywords:** search for meaning, presence of meaning, readiness for political participation, cluster analysis, person-oriented approach

## Abstract

This study explored the association between meaning in life and readiness for political participation based on meaning-in-life profiles among Chinese late adolescents. A total of 1030 college students (mean age = 19.69 ± 1.47 years) in Hong Kong participated in this study. First, we used a cluster analysis to investigate meaning-in-life profiles based on two dimensions: “presence of meaning” and “search for meaning”. Furthermore, we investigated the association between meaning profiles and readiness for political participation. Results revealed three distinguishable profiles, which emerged in both male and female adolescents. Students with “high-presence” and “high-search” attributes and students with “low-presence” and “high-search” characteristics showed greater readiness to engage in normative and non-normative political actions than did those with a “high-presence” and “low-search” profile. Our research fills the research gap on meaning profiles in Asian adolescents and provides the empirical basis for an alternative account of youth political participation.

## 1. Introduction

Meaning in life is usually conceptualized as coherence, significance, and purposefulness in life which is a significant predictor of human functioning (e.g., [1,2]). In his seminal work *Man’s Search for Meaning*, Frankl [1] argued that human beings have an innate motivation to quest for meaning in their lives while meaning in life can be derived from every moment of living, including pleasant and suffering experiences. People are able to see love and sacrifice even in the suffering, which offer them significant meaning to endure the hardship and conduce to their psychosocial functioning. Steger and colleagues [3] further contended that meaning in life is a broad concept consisting of two distinct dimensions: presence of life meaning as well as search for life meaning. Much effort has been made to understand how “having” meaning and “seeking” meaning contribute to different aspects of well-being and mental health [3,4,5]. However, these studies mainly focus on outcomes indexed by internal psychological states, while little is known about how these two meaning dimensions influence social attitudes and behavior such as political participation.

This study investigated the relationship between meaning in life and readiness for political participation. We argue that search for meaning possibly paves the way for one’s political participation, because participation in activities that address issues of public concern serves as a potential means for individuals to find their meaning and purpose in life. This relationship may be particularly salient in late adolescence when young people often seek meaning by questioning and defining their positions in the world [6], while political participation can be a vehicle for such meaning exploration [7].

In this study, we examined this relationship via a person-oriented approach, because search for meaning can be accompanied by presence of meaning or absence of meaning [8]. This approach allows us to identify different patterns of meaning in life in individuals and further explore the relationship between different meaning profiles and readiness for political participation. We targeted late adolescents in Hong Kong in the aftermath of the social movement against the Hong Kong government’s intention to amend the Extradition Law (known as the “anti-extradition bill movement”), because young people in Hong Kong demonstrated unprecedented activism in this social movement [9,10]. Overall, this study will not only extend our understanding of the effect on meaning in life to the political domain but also provide a new framework to look at youth political participation.

### 1.1. ”Presence of Meaning” and “Search for Meaning”

Frankl [1] argued that human beings generally have a “will to meaning” which drives them to find out the unique meaning of their lives, while Steger, Frazier, Oishi, and Kaler [2] considered it important to conceptually differentiate two dimensions of meaning in life—presence of meaning (i.e., having meaning) and search for life meaning (i.e., seeking meaning). These two dimensions represent different aspects of meaning in life but they are not necessarily situated at opposite ends of the continuum. Presence of meaning pertains to the experience of meaning whereas search for meaning concerns the process of meaning exploration. Specifically, while presence of meaning refers to the degree to which people perceive their lives as significant, valuable, and purposeful, search for meaning refers to the degree to which people intend to find or increase their life meaning, significance, or purpose [3]. In other words, people with high levels of presence of life meaning feel that their lives are very meaningful, while people with high levels of search for life meaning work hard to find out how they can live more meaningful lives [11].

The association between “presence of meaning” and “search for meaning” is complex. Conceptually, Frankl [1] portrayed search for life meaning as an innate and healthy process leading to increased presence of meaning, whereas Baumeister [12] regarded it as a response evoked by a crisis of meaning. Substantial studies based on Western people (including late adolescents) showed that these two dimensions were negatively related [2,11]. These findings suggest that people are more likely to search for meaning when they are devoid of meaning. However, studies from the Asian cultures indicate a different picture (e.g., [13,14]). For example, Steger, Kashdan, Sullivan, and Lorentz’s [11] study found a positive relationship between the two in a Japanese adult sample. Most studies in Chinese societies reported positive relationships (e.g., [13,14,15]) or non-significant relationships (e.g., [5,16]).

Steger et al. [17] attempted to explain the cultural difference in search for meaning from cognitive style and values perspectives. The dominant way of cognitive style in many Western cultures is portrayed as analytic, which focuses on the attributes of individual objects without reference to their contexts [18]. In contrast, the Asian cognitive style is characteristic of dialectical or holistic thinking, which integrates individual objects with their contexts [19]. Central to dialectical thinking is the acceptance and reconciliation of contradiction, as well as the expectation of change. Therefore, different from Western people who tend to highlight distinction, reject the coexistence of opposites, and expect constancy in rules, Asian people tend to engage in contradiction by seeking a “middle way” and to assume the state of the world to be dynamic [18]. Accordingly, the two seemingly contradictory dimensions of meaning in life are more likely to co-exist in Asian culture. The instability of presence of meaning also makes Asian people keep working at life meaning to sustain it [11].

From the cultural values perspective, while the Western individualist culture highlights self-enhancement (i.e., motive of enhancing good feelings about oneself and maintaining positive self-view), the Asian collectivist culture emphasizes self-improvement (i.e., motive of striving for a better and more socially recognized self [20]). Search for meaning represents a continuous self-improvement endeavor of creating or deepening one’s meaning in life, which may not be necessarily driven by meaning deficiency (see also [5,14]). Despite the possible cultural variations, both positive and negative associations found in previous studies were moderate at most. It suggests that these two aspects of life meaning are not entirely opposite or identical, and different people may have different combinations of the two dimensions.

### 1.2. Understanding Meaning in Life with a Person-Oriented Approach

The person-oriented approach is a typological methodology that enables us to investigate how the two meaning dimensions interact within individuals [8]. In contrast to the variable-oriented approach focusing on the direction and strength of correlational relationships among variables, the person-oriented approach concentrates on recognizing derived patterns among multiple variables based on similarities of characteristics between respondents [21]. In this case, we can identify distinct groups of individuals who share similar scores on the two dimensions of life meaning and further examine group differences in psychological processes and outcomes.

Past studies targeting different populations have shown that individuals can be distinguished by configurations of presence of meaning and search for meaning (e.g., late adolescents [8,22,23] and elderly people [24]). Meaning configurations are likely to unfold in line with identity status [8]. Theoretically, Steger, Frazier, Oishi, and Kaler’s [2] conceptualization of presence of and search for life meaning are consistent with the dimensions of exploration (active and inquisitive search and experimentation) and commitment (confirmation of a choice) in Marcia [25] identity status theory. Marcia [25] identified four types of identity status: (1) achievement, which refers to a state with commitment made through thoughtful exploration; (2) moratorium, which indicates high levels of exploration without commitment; (3) foreclosure, which pertains to commitment achieved without much prior exploration; and (4) diffusion, which refers to a lack of commitment and systematic exploration. With reference to this typology, individuals may be at a stage of meaning achievement (high presence of and high search for meaning), meaning moratorium (low presence of and high search for meaning), meaning foreclosure (high presence of but low search for meaning), or meaning diffusion (low presence of and low search for meaning [2]). Previous studies using samples of college students partially support this conceptual framework. Studies from the United States [8] and Romania [23] yielded five clusters, including one additional “undifferentiated cluster” with scores of both dimensions at the average level. Another study based on Polish college students found only three clusters, with the meaning diffusion category absent [22].

So far, little is known about how these two meaning dimensions interplay on individuals in Asian culture, where the coexistence of presence of meaning and search for meaning is advocated [11,15]. One study [21] based on Chinese adolescents explored the profiles of purpose in life (i.e., an enduring and benign tendency to do something meaningful to the self and the world [26]), which is similar to but distinct from meaning in life. This study found four purpose profiles corresponding to four meaning statuses: achieved, uncommitted, foreclosed, and diffused. Therefore, it is likely that four clusters of meaning in life will appear in late adolescents from the Asian collectivist culture.

### 1.3. Meaning in Life and Readiness for Political Participation in Late Adolescence

With cognitive maturity and increased interaction with the social world, adolescents start to explore life possibilities and future directions [6], and such activities become more salient during late adolescence, a transitional period to adulthood [23]. This life stage is also called emerging adulthood by Arnett [27]. Different from a “full-fledged” adult, individuals from late teens to early twenties have not yet stabilized their work and life and continue to explore their identities and possible life directions [28,29]. Parallel to identity exploration is youth’s creation of meaning in life [23]. Steger, Oishi, and Kashdan [3] found that participants in late adolescence reported the strongest desire and efforts to search for meaning when compared with participants in other stages of adulthood. As life meaning covers understanding of the self and the world as well as one’s relationship with the world [30], young individuals who want to search for meaning may prefer to engage in public affairs and contribute to a larger world, which possibly offers them an answer about their positions in the world.

We argue that the search for meaning is closely related to readiness for political participation because political participation provides individuals with four major sources of meaning in life: sense of belonging, enhancement of self-esteem, perceived sense of control, and sense of symbolic immortality [30,31]. Political participation is defined as actions undertaken to influence government or politicians to implement a change in decisions about social issues [32,33]. First, this is a process enabling individuals to connect with others or groups sharing a similar political ideology and a common goal [34]. Such connections define their role in the community, which will promote their sense of meaning. Furthermore, belonging and identification with a social group can promote self-esteem, sense of control, and symbolic immortality [35,36]. Individuals’ self-esteem is promoted when they are accepted by a larger group [36], and then, they can feel that their lives are more worth living. Group identification further strengthens one’s sense of control such as capability of attaining desired outcomes [37]. A heightened sense of control makes people perceive their lives to be more predictable and thus more meaningful. Lastly, according to the terror management theory [38], fighting for one’s values and perspectives reduces one’s anxiety due to a deficit of life meaning, which eventually promotes one’s symbolic immortality.

Empirically, Lin [31] showed that participants who thought more about life meaning and purpose of life reported higher levels of political participation. Scales et al. [39] also found that young people (12–24 years) who engaged in more attempts to discover meaning were more likely to undertake voluntary service—another form of civic engagement which enables people to contribute to the welfare of others. Altogether, we predicted that late adolescents with higher levels of search for meaning would develop more favorable attitudes toward political participation. Specifically, individuals in meaning achievement and moratorium statuses were expected to demonstrate stronger readiness for political participation than those in meaning foreclosure and diffusion statuses.

However, the aforementioned studies did not consider the presence of meaning in the linkage between search for meaning and civic engagement. Prior person-oriented research (e.g., [8,22,24]) has shown that search for meaning can be accompanied by different levels of presence of meaning. People searching for meaning with substantial life meaning (i.e., meaning achievement) usually demonstrate the highest level of well-being and mental health, while those searching without life meaning (i.e., meaning mortarium) often show the lowest or second-lowest level among people of the four statues (e.g., [8,22]). Individuals with meaning achievement probably seek meaning for personal growth (e.g., doing something to enrich their identified meaning in life), while individuals with meaning mortarium probably seek meaning to relieve internal turmoil and distress (e.g., exploring something to help them out of meaning crisis [8]). It is still unknown whether these two kinds of search enhance people’s readiness to take up political causes.

This question is better understood when two types of political participation (i.e., normative and non-normative participation) were differentiated. Normative political actions are usually undertaken with adherence to existing laws and regulations in a given society [40], such as voting, signing petitions, and engaging in peaceful demonstrations. These behaviors are usually regarded as socially acceptable and developmentally appropriate for youth [41]. In contrast, non-normative political participation includes illegal and probably radical actions, such as violent protest and forcibly occupying a government institute [42]. These political actions are socially controversial. They likely present risks to young people’s personal safety and psychological well-being [43] and probably indicate a problematic developmental experience [42]. Therefore, late adolescents with meaning achievement may opt for normative political participation, while those with meaning moratorium may opt for non-normative political participation more than those with other statuses.

### 1.4. The Current Study

The current study focused on the presence of and search for meaning in life as factors that might influence late adolescents’ readiness for political participation. We adopted a person-oriented approach using a cluster analysis because it informs differentiated configurations of meaning dimensions within individuals. We targeted college students in Hong Kong for two primary reasons. First, college students represent late adolescents in Hong Kong, because approximately nine-tenths of high school graduates enter into tertiary education institutions, with 37.5% of them enrolled in degree programs and 52.0% in sub-degree (or non-degree) programs such as associate degrees and higher diplomas [44]. This study included students from both degree programs and sub-degree programs. Second, Hong Kong witnessed a fierce “anti-extradition bill movement” in 2019, and college students took a strikingly active role in this social movement [9]. Meaning creation is a critical developmental task during late adolescence [8,23]. Thus, it is important to understand why some late adolescents are willing to take up political causes even at the expense of their personal interests from their internal meaning states.

We first identified the meaning in life profile among college students in order to fill a gap of meaning profile in the Asian collectivist culture. According to the conceptualization of meaning in life [2] and previous studies (e.g., [8,21]), we expected four clusters to emerge among late adolescents, corresponding to four meaning statuses: (1) achievement (i.e., high presence, high search), (2) moratorium (i.e., low presence, high search), (3) foreclosure (i.e., high presence, low search), and (4) diffusion (i.e., low presence, low search) (Hypothesis 1). As Chinese culture accepts the coexistence of contradiction and upholds the search for meaning as an enduring effort of self-improvement [5,11], we also expected the largest group to comprise late adolescents of achievement status (Hypothesis 2).

Next, we examined the relationship between meaning profile and readiness for political participation as a way to differentiate more politically ready profiles of meaning. As search for meaning was positively related to civic engagement [31,39], we hypothesized that higher readiness levels for political participation in the clusters would be associated with higher levels of search for meaning. Furthermore, as political participation can be classified as normative and non-normative forms, we expected the highest readiness for normative political participation to emerge in the cluster representing meaning achievement (Hypothesis 3b), while the highest readiness for non-normative political participation to emerge in the cluster representing meaning moratorium (Hypothesis 3c). We did not have a hypothesis about the difference between foreclosure and diffusion in the level of readiness for political participation, because there is no evidence suggesting that meaningfulness relates to political participation (or intention). The hypotheses are presented in Table 1.

## 2. Materials and Methods

### 2.1. Participants and Procedure

The data were retrieved from an online survey about Hong Kong college students’ political attitudes conducted in 2020 when the large-scale “anti-extradition bill movement” almost ceased. Participants were 1033 college students who held Hong Kong permanent residency, including 602 students from degree programs and 431 students from sub-degree programs (i.e., associate degrees and higher diplomas). The average age was 19.69 ± 1.47 years, and 63.9% were females. Participants were invited to complete an online survey in Qualtrics—a widely used platform that enables researchers to administer and manage online surveys. Initially, 1141 college students completed the survey. However, we removed 108 unqualified cases (9.5% of the cases), including those enrolled in overseas institutions, with ages over 25 years (i.e., beyond late adolescence or emerging adulthood [28]), and failing to answer the two attention-check questions correctly. To ensure data quality, we used two instructional response items to check participants’ attention. Following the practices of previous research (e.g., [45]), we asked the participants to choose a specific option (e.g., “This is an attention check. Please select ‘strongly disagree’ for this item”). More participant demographic information can be found in Table 2.

This study had received ethical approval from the first author’s institution before its commencement. Participants all provided informed consent prior to the survey. As a token of appreciation, they received a supermarket coupon of HKD 50 (approximately USD 6.41) upon the completion of the survey.

### 2.2. Measures

Participants reported on a battery of scales assessing their meaning in life, political attitudes, and demographic background. We presented all the materials in Chinese.

#### 2.2.1. Meaning in Life

We used the Meaning in Life Questionnaire (MLQ [2]) to assess the two dimensions of meaning in life. The participants indicated their agreement to five items about presence of meaning (e.g., “I understand my life meaning”) and five items about search for meaning (e.g., “I am searching for meaning in my life”) on a 7-point Likert scale (1 = strongly disagree; 7 = strongly agree). This scale has been translated into different languages, with its Chinese version being used frequently in different Chinese populations (e.g., [5,13,14,16]). The current study showed good internal consistency of the two subscales. We computed the average scores for presence of meaning and search for meaning, respectively.

#### 2.2.2. Readiness for Political Participation

Participants’ readiness for participating in political actions was measured by 11 items derived from the studies of Šerek, Machackova, and Macek [42], as well as Dahl and van Zalk [32]. Participants reported on their likelihood of taking seven normative political actions (e.g., “sign a petition”) and four non-normative political actions (e.g., “together with others, forcibly occupy some administrative or governmental building”) when they think something bad is happening in society using a 6-point scale (1 = definitely will not; 6 = definitely will). We translated and back-translated these items by following the recommended practice [46]. The current study showed good internal consistency of the two subscales. We computed the average scores for readiness for normative political participation and readiness for non-normative political participation, respectively.

#### 2.2.3. Controlled Variables

We controlled participants’ demographic background as well as their political interests in this study, as previous studies suggested that these factors might influence political participation or attitudes (e.g., [31,42]). First, participants provided information about their gender, age, monthly household income, parents’ educational levels, and study program. Participants also reported their monthly household income in Hong Kong dollar on 11 levels (1 = below HKD 10,000; 11 = HKD 100,000 or above). They further reported on their fathers’ and mothers’ education levels on six levels (1 = primary education or below; 2 = junior secondary education; 3 = senior secondary education; 4 = tertiary education-sub-degree programs; 5 = tertiary education-degree program; 6 = master’s degree or above). Second, participants indicated their degree of interest in political issues by one single item [47] on a 5-point Likert scale (1 = not interested at all; 5 = very interested).

## 3. Results

### 3.1. Meaning in Life Profile

We used a cluster analysis to analyze the data on the two dimensions of meaning in life. Cluster analysis is an analytic tool that can identify naturally occurring profiles based on presence of meaning and search for meaning, and it allows researchers to derive patterns from the data in an exploratory manner [8,23]. Given the limited evidence of meaning profiles in Chinese population, it is suitable to use cluster analysis in our current study. In the first step, we standardized both variables within the total sample. We performed a hybrid of hierarchical clustering and k-means clustering through the R program factoextra package [48]. This hybrid algorithm has been recommended by many studies (e.g., [8,24]) because it integrates the advantages of both hierarchical clustering and k-means clustering [49]. Hierarchical clustering allows researchers to quickly recognize the structures within data without initial assumption, and k-means clustering allows reassignment of cases to optimize the cluster membership by minimizing the within-cluster variance and maximizing between-cluster variance based on the initial seed provided by hierarchical clustering [8]. This algorithm consists of three steps. First, a hierarchical cluster analysis was conducted based on Ward’s method and squared Euclidian distances [50], and cuts the tree into k-clusters. Second, it computes the center of each cluster. Finally, it conducts a k-means clustering by using the set of cluster centers in the second step as initial cluster centers and then optimizes the clustering.

**Figure 1:**
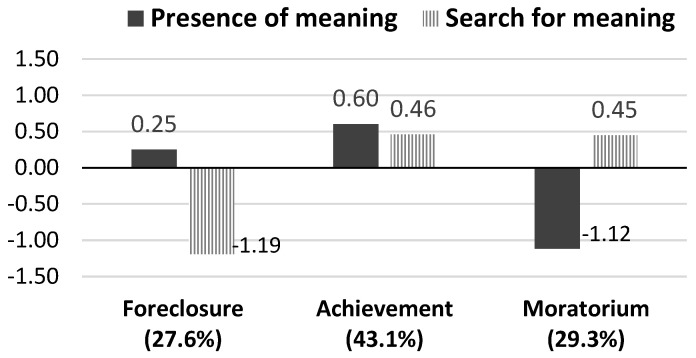
The centers of clusters. Note. This figure presents the standardized scores with mean as 0 and one standard deviation as 1.

We tested two- to six-cluster solutions and compared their Calinski–Harabasz (CH) index [51] and the proportion of explained variance by the cluster solution (η^2^) [52]. First, the CH index suggested that three-cluster solutions provided the better fit (two- to six-cluster solutions: 499.34, 608.65, 563.85, 558.46, 579.96). Second, although the amount of explained variance increased with the number of clustering (.33, 0.54, 0.62, 0.68, 0.74), the increase from the two-cluster solution to the three-cluster solution was the largest (21%). Therefore, we adopted the three-cluster solution as the final cluster solution. Figure 1 shows the center (i.e., mean) of each cluster, which indicates how far the cluster deviates from the total sample mean and from the means of the other two clusters [53]. We adopted standardized scores, with distances among the clusters’ means manifested in terms of standard deviation (SD), which can be interpreted as an effect size index [8]. According to the criteria of Cohen’s d (i.e., 0.2 = small effect; 0.5 = moderate effect; 0.8 = large effect [54]), the current cluster centers revealed medium to large deviances from the overall sample mean, indicating substantial differences between the three clusters.

The cluster of “meaning foreclosure” included 285 college students who reported high levels of presence of meaning but very low levels of search for meaning; the cluster of “meaning achievement” included 445 students who reported both high levels of presence of meaning and search for meaning, and the cluster of “meaning moratorium” included 303 students who reported very low levels of presence of meaning but high levels of search for meaning. Different from previous studies among late adolescents [8,23], the cluster including low levels of presence of meaning and search for meaning (i.e., meaning diffusion) was absent.

Two sets of analysis of variance (ANOVA) were performed to test the differences of presence of meaning and search for meaning by cluster. As shown in Table 3, significant mean differences were found in the clusters. Specifically, the achievement cluster showed the highest level of presence of meaning, followed by the foreclosure cluster, which was higher than the moratorium cluster. The achievement cluster and moratorium cluster showed higher levels of search for meaning than the foreclosure cluster, but these two clusters did not differ from each other.

To test the robustness of the meaning-in-life profile, we conducted two independent cluster analyses in the male group (*n* = 317) and female group (*n* = 656). In the male group, the CH index suggested that the six-cluster solution provided the better fit (CH = 215.74), followed by the three-cluster solution (CH = 213.37). Nevertheless, the increase of variance explained by cluster solution from two-cluster solution (.30) to three-cluster solution (0.53) was the largest (23%). Additionally, if the six-cluster solution was adopted, the number of participants in some clusters became too small (<10%). Hence, the three-cluster solution appeared to be most reasonable. Similar to the results of the whole sample, the three clusters represented the foreclosure group (*n* = 103), achievement group (*n* = 169), and moratorium group (*n* = 99). In the female group, the CH index suggested that the three-cluster solution provided the best fit (CH = 389.59), followed by the four-cluster solution (CH = 375.56). The increase of variance explained by the cluster solution from the two-cluster solution (0.32) to the three-cluster solution (0.54) was the largest (22%). Therefore, we considered the three-cluster solution as an appropriate choice. The three clusters represented the foreclosure group (*n* = 188), achievement group (*n* = 273), and moratorium group (*n* = 195). Additional results of this robustness test can be found in the Supplementary Materials (see Table S1 and Figure S1). Overall, Hypothesis 1 was not completely supported, but Hypothesis 2 was supported.

We performed a multinomial logistic regression analysis to examine if demographic variables (i.e., gender, age, monthly household income, parents’ educational levels, and study program) influenced the cluster membership via IBM-SPSS software. The categorical variables of gender (0 = female, 1 = male) and study program (0 = sub-degree program, 1 = degree program) were dummy coded. To avoid multicollinearity of socioeconomic indicators, we computed standardized z scores for household income, father’s educational level, and mother’s educational level. Additionally, as paternal education and maternal education were highly correlated (r = 0.61, *p* < 0.001), we took a mean score of these two variables to indicate parents’ educational level. The foreclosure cluster was treated as the reference group. The likelihood ratio tests showed that none of the demographic variables had significant effects on the cluster membership of meaning profile (χ^2^s(2) < 5.24, ps > 0.05). However, further comparisons found that if students were female, they would be more likely to fall into the moratorium cluster, as compared with the foreclosure cluster (b = 0.39, SE = 0.19, Exp(B) = 1.48, *p* < 0.05). The estimated coefficients are presented in the online Supplementary Materials (see Table S2).

### 3.2. Relationship with Readiness for Political Participation

Two separate sets of analysis of variance (ANOVA) were computed to test the effect of meaning profile and readiness for (normative vs. non-normative) political participation. Cluster membership was used as a fixed variable and readiness for political participation as a dependent variable. The results are presented in Table 3. First, we found significant differences between clusters in both normative and non-normative political participation. Second, we used Tukey’s honestly significant difference (HSD) post-hoc test to further examine the mean differences between the clusters. Results showed that college students in the achievement cluster (mean difference = 0.28, SE = 0.07, *p* < 0.001) and students in the moratorium cluster (mean difference = 0.23, SE = 0.07, *p* < 0.01) reported higher willingness to engage in normative political actions than did those in foreclosure cluster. However, no significant difference was found in the two clusters with higher levels of search for meaning (mean difference = 0.05, SE = 0.07, *p* > 0.05). Similarly, college students in the achievement cluster (mean difference = 0.27, SE = 0.10, *p* < 0.05) and students in the moratorium cluster (mean difference = 0.25, SE = 0.10, *p* < 0.05) reported higher willingness to engage in non-normative political actions than those in the foreclosure cluster, but there was no significant difference between the clusters with higher levels of search for meaning. Therefore, the current findings supported Hypothesis 3a only.

To increase the robustness of the results, we performed two sets of analysis of covariance (ANCOVA) by controlling the demographic variables (i.e., gender, age, monthly household income, parents’ educational levels, and study program) and political interest. As mentioned above, we used the dummy coded variables of gender, study program, standardized scores of income and parents’ educational levels. Results of readiness for normative political participation remained largely the same. Although students’ age (F(1, 913) = 4.40, *p* < 0.05, η^2^ = 0.01), parents’ education (F(1, 913) = 4.73, *p* < 0.05, η^2^ = 0.01), and political interest (F(1, 913) = 346.26, *p* < 0.001, η2 = 0.28) influenced the readiness for normative political participation, cluster differences still existed. Pairwise comparison using Bonferroni correction showed that college students in the achievement cluster (mean difference = 0.15, SE = 0.06, *p* < 0.05) and students in the moratorium cluster (mean difference = 0.18, SE = 0.07, *p* < 0.05) reported higher willingness to engage in normative political actions than those in the foreclosure cluster. Similarly, although political interest (F(1, 913) = 199.63, *p* < 0.001, η^2^ = 0.18) positively influenced the non-normative political participation, cluster differences remained true. College students in the achievement cluster (mean difference = 0.24, SE = 0.09, *p* < 0.05) and students in the moratorium cluster (mean difference = 0.28, SE = 0.10, *p* < 0.05) reported higher willingness to engage in normative political actions than those in the foreclosure cluster.

## 4. Discussion

Meaning in life has been widely acknowledged as a cornerstone of well-being and optimal human functioning [1,2]. This study aimed to understand late adolescents’ experience of meaning in life based on two dimensions (i.e., presence of and search for life meaning) and relate their meaning experience to readiness for political participation. By using a person-oriented approach (i.e., cluster analysis), this study found three distinct meaning patterns based on the two meaning dimensions—meaning achievement, meaning foreclosure, and meaning moratorium. Furthermore, this study found that meaning-searchers were more inclined to participate in political activities. These findings provide insights into understanding the complex relationship between the presence of meaning and the search for meaning within individuals in Asian culture and offer initial evidence from Chinese youth suggesting that political participation (intention) can be understood via the lens of meaning in life.

### 4.1. Meaning Clusters

This is the first study that has examined the person-centered patterns of meaning in life among people from Asian cultures. Consistent with Krok’s [22] study but different from other studies using samples of late adolescents [8,23], we found three clusters representing meaning achievement, meaning foreclosure, and meaning moratorium. The largest cluster (43.1%) comprised individuals who showed both high levels of presence of meaning and search for meaning. Echoing Frankl’s [1] original conceptualization of search for meaning, this cluster represents young people who are seeking meaning for growth. Search for meaning accompanied by presence of meaning is not only accepted but also highly encouraged in Asian culture because it indicates one’s persistent effort toward self-improvement [5,11]. This is particularly in line with the Confucian ideal of self-cultivation (xiu xin)—a constant effort to promote development of mind and body and reach spirituality and moral perfectibility [55]. Chu and Fung [15] coined such search as “growth search,” which occurs when individuals are striving to deepen or enrich their understanding of life meaning under positive circumstances. During late adolescence or emerging adulthood, young people may have a certain understanding of their life but continue extending their understanding and work hard to reach their identified meaningful goals. For example, they may have experienced a great deal of meaningfulness from their family, and thus, they try to find out how they can make their lives more meaningful through contributing to their families. As this study did not measure value on self-improvement or Confucianism, we encourage future studies to extend the understanding on cultural variation in relationship between the two meaning dimensions by considering the moderating role of culturally specific values.

The second largest cluster (29.3%) comprised individuals who searched for meaning with an absence of meaning. It fits the description of the search for meaning as a natural response to an absence of meaning [11,12]. Moreover, consistent with the meaning-making theory [56], when a global meaning system (e.g., beliefs, goals, and sense of meaning) is threatened or dampened by a stressful situation, people tend to engage in a meaning-making process to restore and re-construct their meaning. This search is congruent with Chu and Fung’s [15] conception of deficiency search—a struggle for filling in the void of meaning and a desire to diminish the tension between expected meaning and deficiency of it under negative conditions. This group of young people probably struggle for life meaning because they have not had a clear understanding of it or their original life meaning has been challenged. The last cluster (27.6%) comprised young people who were satisfied with their life meaning and did not engage in meaning searching. Similar to the moratorium cluster, it is also in line with the theories that describe the negative relationship between presence and search [11]. This group of people did not work at meaning anymore because they considered their lives meaningful enough.

We did not observe the diffusion group in this study. Late adolescents are generally active in meaning searching [3,28]. Additionally, Chinese culture probably strengthens young people’s motivation to search for meaning, as prolonged meaning quest represents an endeavor of self-improvement—making a better self and a better life [5,11,57]. Therefore, Chinese late adolescents tend to engage in meaning search when their presence of meaning is endangered. Another possibility concerns the time point of data collection. The anti-extradition bill movement brought about tremendous social impacts on Hong Kong people and the society as a whole, which presumably challenged youths’ original meaning systems. Under this situation, it is rare to find youth who are perplexed about life meaning but devoid of motivation to search for it.

Overall, our study has lent partial support to Steger, Frazier, Oishi, and Kaler [2] original classification of meaning status. To further understand whether the current classification is due to cultural factors or social events, we recommend future studies replicate the findings in other Asian samples and relate cultural factors (e.g., dialectical thinking and self-improvement motivation) to the classification.

### 4.2. Meaning Clusters and Readiness for Political Participation

Our study further revealed that individuals in the clusters with a high level of search for meaning reported higher readiness for political participation in both normative and non-normative forms than those in the cluster with a low level of search. After controlling for participants’ demographic backgrounds and political interest, this difference remained true. Consistent with our expectations and previous studies [31,39], the search for meaning may motivate people to engage in public affairs that concern collective welfare. Despite having emotional costs and physical risks [58], young meaning-searchers may expect to strengthen their identification with a certain political group, obtain a sense of empowerment, and at least get opportunities to express and uphold their ideologies and values during political participation [31]. All of these outcomes may enhance their meaningfulness. In general, political participation is a vehicle to connect individuals with something larger than themselves, which fits the objective of meaning searching. Therefore, they are more willing to get involved in it when they perceive something going wrong in society. However, it is noted that political participation is not the single approach to reaching meaningfulness. According to the pragmatic meaning-regulation approach [59], individuals’ behavioral selection to fulfill their quest for meaning is contingent on their values and perceived effectiveness of the activity. For example, one study found that people who prioritized environmental wellness over economic interest tended to engage in pro-environmental activities when they wanted to search for meaning [31]. Considering there are multiple paths to reach meaningfulness, future studies can explore more behaviors that help late adolescents to obtain their meaning in life.

We did not find differences between the meaning achievement group and the meaning moratorium group in their attitudes toward political participation. Both showed a high level of willingness to take up normative and non-normative political actions. This may be due to the special social atmosphere and sentiment during the “anti-extradition bill movement” [10]. Although non-normative political participation is against laws and social regulations, many Hong Kong people demonstrated high tolerance of illegal political actions against government and police in this social movement [60]. These illegal political activities, such as violent confrontation with police, were thought to be conducted to combat social injustice, despite its incalculable costs to socioeconomic development [61]. In this situation, young meaning-searchers are more willing to participate in both legal and illegal actions, regardless of their level of presence of meaning. Future studies are needed to examine how the “normalization” of non-normative political actions influences meaning-searchers’ readiness for political participation. Another possibility is that young people may not have sufficient cognitive and social competence to differentiate normative and non-normative political participation, including the impacts on themselves and society. Future research is encouraged to explore whether cognitive and social competence will influence young meaning-searchers’ choice between normative and non-normative political action. Given a lack of evidence-based cognitive and social competence education for youth in Hong Kong [10,62], we suggest educators and youth work professionals to think more about how to design and implement corresponding educational programs that enable youth to differentiate normative and non-normative political actions.

The current findings add to the literature explaining why some young people are more willing to take up political actions than others. Political participation is a complex process subject to a variety of factors, ranging from personal (e.g., political interest and efficacy), interpersonal (e.g., social identity), collective (e.g., collective anger), to contextual (e.g., political system [41,63]). Through the lens of meaning in life, this study offers a novel framework to look at readiness for political participation among late adolescents. Admittedly, readiness, which pertains to willingness and inclination, is not equal to participation. Nevertheless, longitudinal studies have shown that readiness for political participation predicted actual political action [64]. Moreover, participants may feel safer to report their true readiness compared with their true behaviors, especially when the behaviors are illegal. An inquiry into attitudes rather than actual behavior helps us widen the coverage of late adolescent population under survey. Overall, this study highlights the possibility to understand adolescent political participation from a meaning-making perspective.

### 4.3. Limitations and Future Directions

The current results should be interpreted with caution. First, the limitation of the current sample constrains the generalization of the findings. Cluster analysis is data-driven, and thus different patterns might emerge from diverse populations. It is important to replicate the cluster solution in other Chinese societies (e.g., mainland China) or other Asian societies (e.g., Japan). Second, with the adoption of a cross-sectional design, the causal relationships between meaning profile and political participation cannot be firmly established. According to the positive youth development perspective, it is theoretically reasonable that one’s developmental assets, including meaning in life, serve as an antecedent of adolescents’ well-being and behavioral functioning [65,66,67,68]. Nevertheless, to understand the direction of relationships, longitudinal designs are needed (see [69]) or an experimental design that manipulates one’s meaning in life (see [70]). Third, the current results relied on participants’ self-report, which might include common method variance that leads to an overestimated correlation among variables. Although self-report is an appropriate method to probe into subjective and abstract concepts such as meaning in life, future studies may consider supplementing the study with a qualitative method to understand people’s meaning in life.

Fourth, this study focused on readiness or behavioral intention rather than actual behavior of political participation. Although this study reveals the late adolescents’ political preference, it is still unknown how meaning-in-life profiles relate to different types of political involvement. The theory of planned behavior regards behavioral intention as the most direct factor affecting behavior [71], but to what extent intention can translate into behavior depends on a variety of personal and environmental factors [72]. Thus, future studies could focus on actual participation. Finally, despite having controlled for political interest, this study did not consider the roles of other motivational factors such as personal values in the readiness for political engagement. Cross-national research has shown that while values of openness to change (e.g., autonomy of thought) and self-transcendence (e.g., universalism) were positively related to political engagement, values of conservation (e.g., conformity) were negatively related to political engagement [73]. Lin’s [31] study has also shown that meaning-searchers tended to engage in political activities when they valued openness to change (vs. conservation). However, these studies did not clearly differentiate normative from non-normative political actions, and thus, little is known whether specific values motivate people to adopt a non-normative approach of political engagement regardless of its potential negative consequence. Future studies are encouraged to examine how personal values influence meaning searchers’ preferences for normative and non-normative political engagement, respectively.

## 5. Conclusions

Going beyond previous research that mainly focuses on how meaning in life contributes to mental functioning, we contend that meaning in life, comprising both “presence of meaning” and “search for meaning”, also relates to late adolescents’ motivation for political engagement. Specifically, we argue that different compositions of the two meaning dimensions reflect different levels of motivations. Thus, via a person-oriented approach, this study firstly identified three distinguished meaning-in-life profiles pertaining to the two meaning dimensions (i.e., meaning foreclosure, meaning achievement, and meaning moratorium) among Chinese late adolescents. Furthermore, we found that people of clusters with higher levels of “search for meaning” showed a higher level of readiness for political engagement.

While acknowledging the above-mentioned limitations, the current study has filled the gap in the literature regarding the nuanced relationship between meaning-in-life dimensions in Chinese population and provided insights into how these constructs may contribute to late adolescents’ readiness for political participation. We highlight search for meaning as a motive for youth political participation. We encourage future studies to understand youth political participation via the lens of meaning in life in different sociopolitical contexts and on different personal conditions.

## Figures and Tables

**Table 1:** Hypotheses of the current study.

Hypothesized Clusters	Hypothesized Level of Readiness for Normative Political Participation *	Hypothesized Level of Readiness for Non-Normative Political Participation *
Achievement	3	2
Moratorium	2	3
Foreclosure	1	1
Diffusion	1	1

Note. * The larger the number the higher the level of readiness.

**Table 2:** Descriptive information of the participants.

Variables	Number (%) ^a^	
Monthly Household Income		
HKD29,999 or below	405 (39.2%)	-
HKD 30,000 to $59,999	379 (36.7%)	-
HKD 60,000 to $89,999	112 (10.8%)	-
HKD 90,000 or above	133 (12.9%)	-
Not know or not report	42 (0.4%)	-
Parents’ education	Father	Mother
Primary education or below	118 (11.4%)	133 (12.9%)
Secondary education	589 (57.0%)	624 (60.4%)
Tertiary education	205 (19.8%)	187 (18.1%)
Master’s degree or above	49 (4.7%)	34 (3.3%)
Not report	72 (0.7%)	55 (0.5%)

Note. ^a^ The responses to a few options were aggregated.

**Table 3:** ANOVAs and post-hoc cluster comparisons.

		Mean (SD)		F	η^2^	Reliability (Cronbach’s α)
	Foreclosure	Achievement	Moratorium			
Presence of meaning	4.30 (0.68) _a_	4.59 (0.46) _b_	3.20 (0.54) _c_	607.70 ***	0.54	0.92
Search for meaning	3.84 (0.66) _a_	5.46 (0.59) _b_	5.44 (0.76) _b_	609.61 ***	0.54	0.88
Readiness for normative political participation	4.06 (0.94) _a_	4.34 (0.83) _b_	4.29 (0.97) _b_	8.82 ***	0.02	0.86
	4.10 (0.94) _a_	4.35 (0.83) _b_	4.31 (0.95) _b_	# 4.12 *	0.01	
Readiness for non-normative political participation	3.01 (1.27) _a_	3.27 (1.25) _b_	3.25 (1.26) _b_	4.42 *	0.01	0.91
	2.98 (1.26) _a_	3.31 (1.26) _b_	3.28 (1.25) _b_	# 6.17 **	0.01	

Note. Different subscripts (_a, b, c_) of mean values indicate significant difference statistically (*p* < 0.05). # Results of ANCOVA. * *p* < 0.05, * *p* < 0.01, *** *p* < 0.001.

## Data Availability

The data presented in this study are available on request from the corresponding author. The data are not publicly available due to privacy.

## Supplementary Materials

The following are available online at https://www.mdpi.com/article/10.3390/ijerph18115765/s1, Table S1: Results of cluster analysis by gender, Table S2: Results of multinomial logistic regression analysis, Figure S1: The cluster centers by gender.
