# Supplementary material for: Meaning-in-Life Profiles among Chinese Late Adolescents: Associations with Readiness for Political Participation
Source: Int J Environ Res Public Health. 2021 May 27;18(11):5765. doi: 10.3390/ijerph18115765 (PMC8198389; doi:10.3390/ijerph18115765)
Supplement: Supplementary file 1 [file ijerph-18-05765-s001.zip › ijerph-1211049-supplementary.pdf]

Supplementary Materials

Table S1. Results of cluster analysis by gender

	**2-cluster**	**3-cluster**	**4-cluster**	**5-cluster**	**6-cluster**
Female					
CH index	307.22	389.59	375.56	363.20	367.75
Variance explained #	.32	.54	.63	.69	.74
Male					
CH index	160.03	213.37	199.68	212.14	215.74
Variance explained #	.30	.54	.62	.70	.75

Note. CH index: the larger the better. # Proportion on of variance explained by the cluster solution.

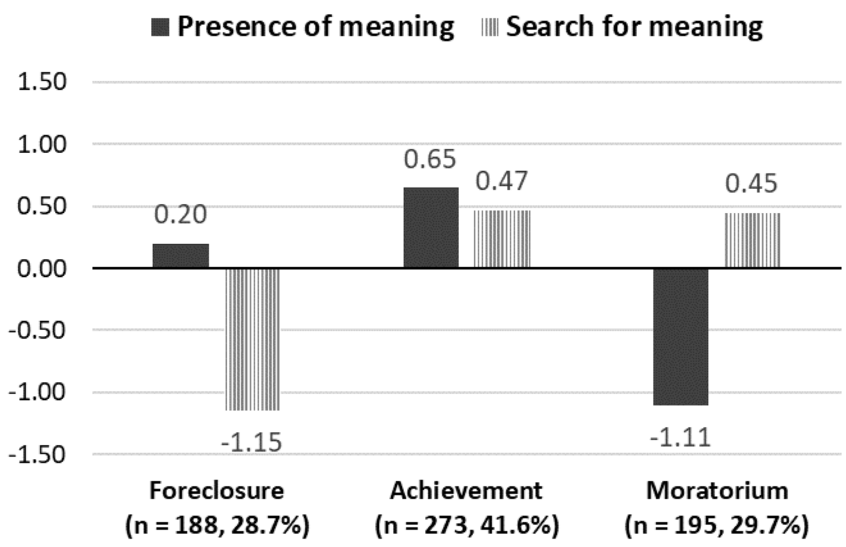

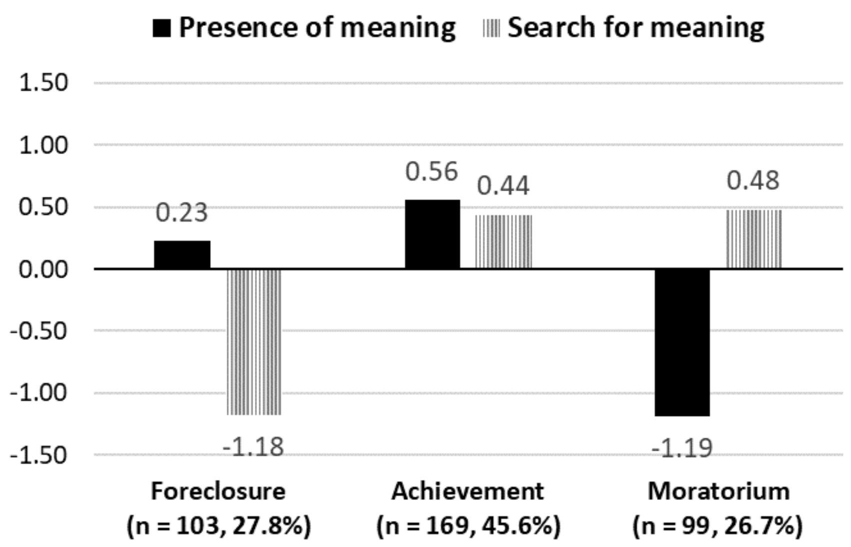

Figure S1. The cluster centers by gender.

Note. Female group (n = 656) is presented on the left and male group (n = 317) is presented on the right.

Table S2. Results of multinomial logistic regression analysis

Cluster		B	Std. Error	Wald	df	Sig.	Exp(B)	95% Confidence Interval for Exp(B)	
								Lower Bound	Upper Bound
Meaning achievement	Intercept	-.526	1.153	.208	1	.649			
	Age	.051	.057	.796	1	.372	1.053	.941	1.178
	Parents' education	-.053	.094	.320	1	.572	.948	.788	1.141
	Income	.089	.091	.954	1	.329	1.093	.914	1.306
	[sex= female]	.259	.169	2.357	1	.125	1.296	.931	1.804
	[sex= male]	0[b]	.	.	0	.	.	.	.
	[program= sub-degree]	-.295	.167	3.116	1	.078	.744	.536	1.033
	[program= degree]	0[b]	.	.	0	.	.	.	.
Meaning moratorium	Intercept	-.263	1.265	.043	1	.835			
	Age	.012	.063	.037	1	.847	1.012	.894	1.145
	Parents' education	-.178	.106	2.844	1	.092	.837	.680	1.029
	Income	-.120	.105	1.318	1	.251	.887	.722	1.089
	[sex= female]	.392	.187	4.371	1	.037	1.480	1.025	2.136
	[sex= male]	0[b]	.	.	0	.	.	.	.
	[program= sub-degree]	-.308	.183	2.845	1	.092	.735	.514	1.051
	[program= degree]	0[b]	.	.	0	.	.	.	.

Note. The reference group is the cluster of meaning foreclosure. Parents' education is a mean score of the Z-score of father's education and mother's education. Income is a standardized Z score.
